# Knowledge and Attitude of School Students About Diabetes Mellitus in the Western Region of Saudi Arabia

**DOI:** 10.7759/cureus.47514

**Published:** 2023-10-23

**Authors:** Mohamed Y Alhilali, Yahya Soliman Alhilaly, Safa Alkalash

**Affiliations:** 1 College of Medicine and Surgery, Umm Al-Qura University, Al-Qunfudah, SAU; 2 College of Community Medicine and Healthcare, Umm Al-Qura University, Al-Qunfudah, SAU; 3 College of Family Medicine, Menoufia University, Shebin Elkom, EGY

**Keywords:** saudi western region, school students, attitude, knowledge, diabetes mellitus

## Abstract

Background: Diabetes mellitus (DM) is a worldwide public health issue. Diabetes has been developing dramatically among young individuals, and childhood onset is now becoming a global epidemic. Data on DM knowledge and attitude among Saudi school pupils in the western region of the country are few. Therefore, this study has been undertaken to assess the level of knowledge and attitude of school students toward DM in the western region of Saudi Arabia.

Methods: A cross-sectional study was conducted on a sample of 850 school students in the western region of Saudi Arabia from October to December 2022. The data were collected using an online questionnaire and analyzed using Statistical Product and Service Solutions (SPSS) (version 23.0; IBM SPSS Statistics for Windows, Armonk, NY).

Results: This study included 850 pupils in total. Females represented most of them (82.1%). The participants' ages ranged from 10 to 18 years, with the majority of them between the ages of 16 and 18. Self-reported diabetes among students was 9.5%, and the most prevalent type was type 1 diabetes. Regarding the physical activity of the participants, 22.6% of them conduct physical exercise for a duration of more than 30 minutes per day, with a significantly higher percentage among non-diabetics, while 34.6% of the diabetics do not exceed 20 minutes of daily exercise, with a P value of 0.017. School students showed a lack of information about symptoms and complications of DM where only diabetics could mention them when compared with non-diabetic students, with P values of <0.001 for each of them. Diabetic students listed DM risk factors, such as genetics, obesity, and smoking, more frequently than non-diabetics; the corresponding P values were 0.004, 0.001, and 0.041. Unfortunately, more than 32% of diabetic students had the misconception that DM is not a controllable disease. According to the majority of diabetic students, soft drinks raise blood sugar levels. The Internet was the main source of information about DM.

Conclusion: The school students' understanding of DM was generally suboptimal. Their understanding of the symptoms, effects, and management of diabetes was low. Most school students in this cohort perceived that DM could not be controlled. Given the high rate of self-reported diabetes in the western region of Saudi Arabia, it is strongly advised to educate children about DM at an early age. Every student at school should adopt a healthy lifestyle that includes a balanced diet and regular exercise, and they should be closely observed by their teachers and parents. Family physicians should regularly check the adherence of diabetic children to their antidiabetic medications and ensure this important point with their caregivers. Psychological assessment and counseling are highly recommended for all diabetic children.

## Introduction

High blood glucose levels are a hallmark of diabetes mellitus (DM), a long-term metabolic disorder that, over time, seriously harms the heart, blood vessels, eyes, kidneys, and nerves [1]. The most prevalent kind of diabetes is type 2, which often affects adults and develops when the body stops producing enough insulin or develops insulin resistance [1]. The prevalence of type 2 DM (T2DM) has significantly increased during the past three decades in nations of all income levels. The pancreas produces little to no insulin on its own in type 1 DM (T1DM). For those with diabetes, having access to affordable medications, especially insulin, is essential. There is a globally agreed-upon target to halt the rise in diabetes and obesity by 2025 [2].

Over the past 30-year period, diabetes has been one of the top drivers of the increasing global burden of disease. A systematic analysis of the global burden of disease (GBD) from 1990 to 2019 across 204 countries has shown that age-standardized disability-adjusted life-years (DALY) rates for diabetes have increased by 24.4% [3]. The International Diabetes Federation, 2022, reported that 537 million adults (20-79 years old) are living with diabetes worldwide (one in 10). By 2030 and 2045, there will be 643 million and 783 million diabetics, respectively [4]. In the Middle East and North Africa, one in six adults (73 million) is living with DM; this number is expected to reach 95 million by 2030 and 136 million by 2045. One in three adults living with diabetes is undiagnosed. DM is responsible for 796,000 deaths in the Middle East and North Africa (MENA) regions in 2021 [4]. The Kingdom of Saudi Arabia (KSA), specifically, is experiencing a high rate of diabetes in its young population [5]. The KSA has the second-highest rate of diabetes in the Middle East and is ranked seventh globally, according to the World Health Organization (WHO) [6]. Nearly three million people are thought to have pre-diabetes, and an estimated seven million people have diabetes [7]. A country's future lies in its youth, who are regarded for their potential as a sort of dynamic human capital that greatly aids in the advancement of the country [8].

The widespread consumption of fast food, fizzy beverages, and energy drinks, as well as a decline in the amount of energy expended through hard labor and regular exercise among children, are major contributors to the development of DM and obesity, as the percentage of children classified as overweight or obese has significantly increased in the past two decades [9,10]. A population-based retrospective study that included children and teenagers aged two to 19 who visited five hospitals and 24 primary care facilities within the National Guard Health System in Saudi Arabia between 2016 and 2021 found a prevalence of overweight (11.2%) or obese (9.4%). Children between the ages of two and six were most likely to be obese (12.3%), and boys were more likely to be obese (10.4%) than girls (8.3%) [11]. In Riyadh, a study of children who had recently been diagnosed with diabetes revealed that 64% of them were overweight or obese; 34% exhibited an indication of insulin resistance, such as acanthosis nigricans; 57% had a family history of the disease; and only 52% had pancreatic antibodies that were positive [12].

Early identification of DM necessitates both medical procedures (e.g., medical history, examination, and laboratory investigations) and patients' involvement (interest) based on how they view the condition (degree of personal health literacy) [13]. The level of health literacy affects people's decisions and actions, which include the ability to choose and access the appropriate form of healthcare [14]. Thus, public knowledge and awareness of diabetes reduce the gaps in diabetes diagnosis and prevent long-term complications among patients with a diabetes diagnosis. Regular monitoring of public awareness of diabetes is necessary to provide effective educational and preventive strategies [15]. Considering the growing incidence of diabetes in the KSA, it is critical to ensure that the population, including children, has sufficient information and awareness of the condition to enable the continued promotion of public health initiatives to limit its prevalence. The primary goal of this research was to assess the knowledge about the disease, its manifestations, and its complications in the western region of Saudi Arabia and to determine whether there are any gaps to bridge. To make the most of the limited resources given to students in schools, this information might be used to improve the existing programs that aim to fill knowledge gaps and dispel misunderstandings.

## Materials and methods

Study design

Between October and December 2022, a cross-sectional survey was carried out to ascertain school students' knowledge about and attitudes concerning DM. The study was conducted in Saudi Arabia's western province. A self-administered, verified electronic questionnaire was utilized to obtain the research data.

Size of the sample

The study was done on a convenient sample of male and female students whose ages ranged from 10 to 18 years old in primary, intermediate, and secondary schools. Based on the total number of school pupils (163,609) in the western region of Saudi Arabia, a minimum sample size calculation of 384 was made, with a 95% confidence interval, an error margin of 5%, and an assumption of good awareness about DM of 50%.

Study setting

The study was carried out in schools in Saudi Arabia's western area, which involves about 26 governorates that are distributed in the two main provinces of Makkah (17 governorates) and Al-Madinah (nine governorates). The total number of schools in the Saudi western region is 1,530, which are distributed in Makkah province (1,148) and Al-Madinah province (382), according to a study from the General Authority for Statistics, KSA [16]. The study sample was chosen using a multistage sampling strategy. In the first stage, the schools were picked from eight randomly selected governorates in the western region: Makkah, Jeddah, Al-Madinah, Al-Qunfudah, Taif, Yanbu, Bahra, and Al-Ardiyat. The second stage involved a random selection of 12 schools from the previously mentioned governorates.

Procedure and tool for data collection

After conducting a literature review of related studies [15,17,18], the study's researchers developed a questionnaire that was used to gather data from schoolchildren of both genders' knowledge and attitudes regarding DM. With the assistance of a three-member expert panel (internal medicine and family medicine), the pertinent questions were designed, grouped inside the questionnaire that was pertinent to the research purpose, and prepared in Arabic as a 33-item questionnaire. The questionnaire was divided into four segments. The first segment includes questions designed to assess the respondents' socio-demographic information, such as age, gender, level of education, and place of residence. The second portion asked about diabetes, whether they had it or not, and those who had it were asked about its type, family history of DM, compliance with its medication, and its impact on their psychological health. The third portion asked about students' lifestyles, nutrition, and exercise histories. The final component assessed their knowledge of diabetes, such as its causes, symptoms, complications, risk factors, and the type of food that affects blood glucose levels. This segment of the developed questionnaire also involved a component focused on their attitudes toward diabetes, its severity, whether it can be controlled, and whether they need additional health education about it.

The survey link was created using the Google Forms application and then propagated among school students by using WhatsApp and Telegram applications of their educational groups and asking them to send the survey link to their peers in the selected schools as a snowball to reach the maximum number of participants. We conducted a pilot study on the first 35 responses (representing about 10% of the sample size) to conduct a pre-test of the survey items in order to determine their understandability by the study group. The estimated average time to fill out the form was three to five minutes. There was no need to change any of the survey's items, and the survey's reliability was assessed with Cronbach's alpha coefficient of 0.81. The data from this pilot study were not included in the main study results.

Data collectors from each governorate helped the study researchers gather the required data as they disseminated the link to this poll via student groups on WhatsApp and Telegram in the schools.

Ethics-related issues

Ethical approval was provided by the Medical Research and Ethical Committee of the College of Medicine of Umm Al-Qura University, Makkah (reference number: HAPO-02-K-012-2022-11-1259). The data were obtained anonymously and kept private. The survey involved an opening question to receive consent from each participant to complete it.

Data evaluation

The primary data were input into Microsoft Excel, and statistical analysis was performed using Statistical Product and Service Solutions (SPSS) (version 23.0; IBM SPSS Statistics for Windows, Armonk, NY). The mean and standard deviation were used to represent quantitative data, whereas frequencies and percentages were used to represent qualitative data. A chi-square analysis was used to examine associations between variables. For small frequency distributions, the exact probability test was applied.

## Results

The total number of students who participated in this study was 850. Most of them were female (82.1%). Our findings demonstrated that most of the study population was aged between 16 and 18 years. Regarding the educational level of respondents, we observed that the highest percentage of them were in secondary school (69.6%), 21.8% of them were in intermediate school, and 8.6% were in primary school. As regards the residential area of students, we noted that the vast majority of them were from Makkah (85.6%) province, and the rest of them were from Medina province (14.4%) (Table 1).

**Table 1:** Demographic characteristics of the participants (n=850)

Variable	Categories	Frequency	Percent
Gender	Male	152	17.9
	Female	698	82.1
Age (in years)	10-12	71	8.4
	13-15	200	23.5
	16-18	579	68.1
Educational level	Primary	73	8.6
	Intermediate	185	21.8
	Secondary	592	69.6
Residential area	Medina province	122	14.4
	Makkah province	728	85.6

**Figure 1:**
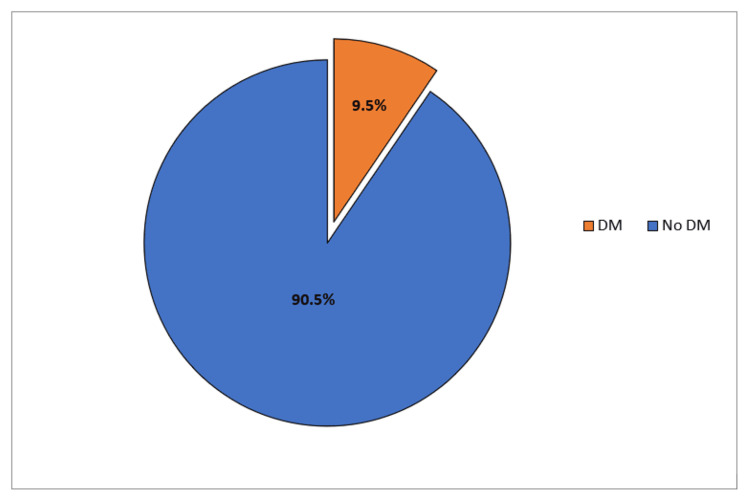
Percentages of self-reported diabetes mellitus among Saudi school students

Our results revealed that self-reported DM among students was 9.5% (Figure 1).

**Figure 2:**
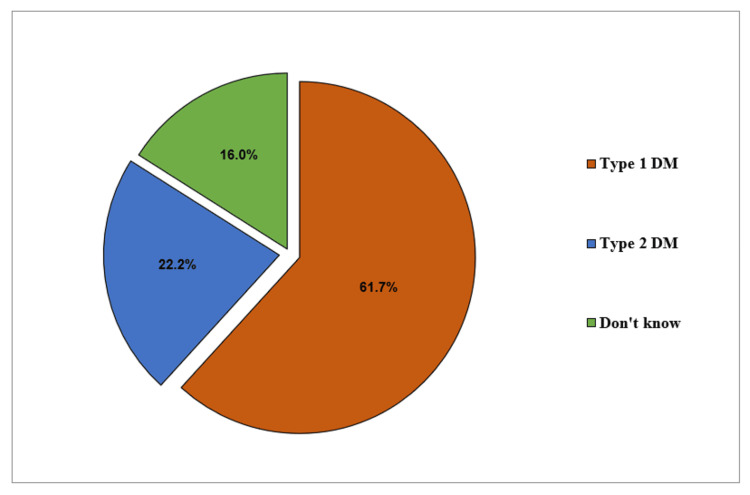
Type of diabetes mellitus among diabetic school students (n=81)

The most prevalent type of diabetes was type 1 diabetes, reported by 61.7%, and type 2 diabetes was reported by 22.2% (Figure 2).

Among the diabetic students, we found that most of them (60.5%) had first-degree family members with diabetes, 39.5% had only one diabetic family member, and 39.5% did not have any. Nearly half of diabetic students have been diagnosed with diabetes before the age of six years; 28.4% have had diabetes since they were 6-12 years old; 11.1% have diabetes at the ages of 16-18 years old; and 8.6% have been diagnosed with diabetes when they were 13-15 years old. Concerning the management of diabetes, the highest proportion of diabetic students revealed they regulated their blood glucose level by injections prescribed by the doctor (40.7%), 29.6% of them used medications and diet together, 13.6% controlled diabetes by diet only, and 16% of them did not care to regulate it. More than half of diabetic students really committed to taking their medications on time and following the diet prescribed by the doctor (58%). Unfortunately, 22.2% of them rarely showed this commitment to medication and diet. Furthermore, most diabetic students stated difficulty sticking to diabetes management in order to regulate their blood sugar, and most of them checked their blood sugar regularly or visited the doctor regularly (Table 2).

**Table 2:** History of diabetes mellitus among diabetic students (n=81)

Variable	Categories	Frequency	Percent
First-degree family history of diabetes mellitus	Yes	49	60.5
	No	32	39.5
Number of family members with diabetes mellitus	One	32	39.5
	Two	8	9.9
	Three	8	9.9
	Four	1	1.2
	Do not have	32	39.5
Age of onset of diabetes mellitus	From 1-5 years old	42	51.9
	From 6-12 years old	23	28.4
	From 13-15 years old	7	8.6
	From 16-18 years old	9	11.1
Having diseases other than diabetes mellitus	Yes	51	63
	No	30	37
The disease other than diabetes mellitus	Cardiovascular diseases	10	12.3
	Hypertension	12	14.8
	Asthma	15	18.5
	I have no disease other than diabetes	30	37
	Do not know	14	17.3
Methods of controlling blood sugar	Injections prescribed by the doctor	33	40.7
	Oral medications and diet together	24	29.6
	Diet regulation only	11	13.6
	Don’t care to regulate it	13	16
Commitment to adhering to the doctor's recommended diet and medication schedule	Always	47	58
	Sometimes	16	19.8
	Rarely	18	22.2
Having difficulty adhering to drugs that regulate blood sugar	Yes	51	63
	No	30	37
Performing regular follow-up visits for diabetes mellitus in the primary healthcare	Yes	51	63
	No	30	37
Diabetes mellitus affects his/her psychological state	Yes	54	66.7
	No	27	33.3

**Figure 3:**
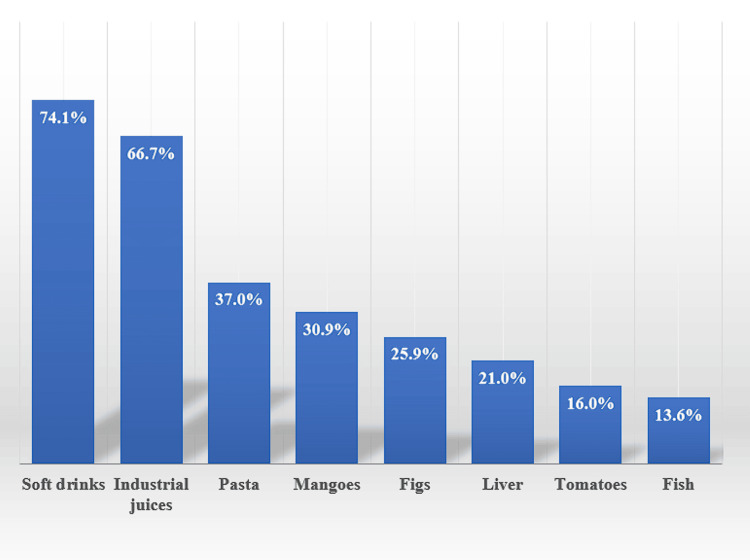
History of different food types that lead to increased blood glucose levels of diabetic students (n=81)

This research showed that 74.1% of diabetic students believed soft drinks to be the most likely food to raise blood sugar levels. Next, 66.7% mentioned commercial juices, followed by pasta (37%), mangoes (30.9%), figs (25.9%), liver (21%), tomatoes (16%), and fish (identified by 13.6%) (Figure 3).

Regarding lifestyle, we found that less than a third of respondents (32.7%) followed a healthy diet regimen, a significantly higher rate among diabetics than those without diabetes (P < 0.001). The highest proportion of participants (38.4%) performed physical exercise such as walking or any other sport once or twice per week; 18.9% performed this exercise three to four times per week; and 12.8% of them performed it five times or more per week. On the other side, 29.9% of respondents did not practice sports, and there was no significant difference between diabetic and non-diabetic students in physical exercise (P = 0.969). When we asked about the duration of exercise, we demonstrated that 22.6% of respondents spent more than 30 minutes, 21.3% spent > 10 and < 20 minutes, 18.0% spent > 20 and < 30 minutes, and 10.8% spent less than 10 minutes on exercise. However, 27.3% did not perform sports, and a higher rate of practice was observed among non-diabetic students (P = 0.017). The results showed that only 37.4% had attended or actively participated in a diabetes workshop, lecture, or educational material, and a higher rate of attendance or active participation was observed among diabetic students (P = 0.005). As regards students' knowledge about DM, 67.2% and 40.3% of non-diabetic students were unable to identify symptoms or complications of DM with P values less than 0.001 for each. Risk factors for DM were correctly mentioned by diabetic students in comparison to non-diabetics, as P values were less than 0.05. Unfortunately, more than 32% of diabetic students had the misconception that DM is a controllable disease (Table 3).

**Table 3:** Comparison between knowledge and attitude toward diabetes mellitus among diabetic and non-diabetic school students # Fischer exact

Variable	Categories	Overall (n=850)	Diabetic students (n=81)	Non-diabetic students (n=769)	P value
	n (%)				
Conducted an assessment of the blood glucose level before	Yes	346 (40.7)	81 (100.0)	265 (34.5)	< 0.001#
	No	504 (59.3)	0 (0.0)	504 (65.5)	
Follow a diet regimen	Yes	278 (32.7)	41 (50.6)	237 (30.8)	< 0.001
	No	572 (67.3)	40 (49.4)	532 (69.2)	
Performing regular physical activities	Yes	596 (70.1)	56 (69.1)	540 (70.2)	0.979
	No	254 (29.9)	25 (30.9)	229 (29.8)	
Frequency of physical activity (Such as walking or any other sport) per week	1-2 times/week	326 (38.4)	32 (39.5)	294 (38.2)	0.965
	3-4 times/week	161 (18.9)	15 (18.5)	146 (19.1)	
	5 times or more/week	109 (12.8)	9 (11.1)	100 (13.1)	
The duration of exercise each time/day	Less than 10 minutes	92 (10.8)	7 (8.6)	85 (11.1)	0.017
	> 10 and < 20 minutes	181 (21.3)	28 (34.6)	153 (19.9)	
	> 20 and < 30 minutes	153 (18)	14 (17.3)	139 (18.1)	
	More than 30 minutes	192 (22.6)	10 (12.3)	182 (23.7)	
Attending or actively participating in diabetes workshop, lecture, or educational material	Yes	318 (37.4)	42 (51.9)	276 (35.9)	0.005
	No	532 (62.6)	39 (48.1)	493 (64.1)	
Diabetes mellitus is a disease caused by a deficiency or resistance to insulin	Yes	580 (68.2)	61 (75.3)	519 (67.5)	0.151
	No	270 (31.8)	20 (24.7)	250 (32.5)	
Symptoms of diabetes mellitus	Polyuria	156 (18.4)	29 (35.8)	127 (16.5)	<0.001
	Polydipsia	129 (15.2)	25 (30.9)	104 (13.5)	
	Loss of weight	41 (4.8)	20 (24.7)	21 (2.7)	
	Do not know	524 (61.6)	7 (8.6)	517 (67.2)	
Complications of diabetes mellitus	Coma	153 (18.0)	13 (16.0)	140 (18.2)	<0.001
	Foot ulcers	66 (7.8)	18 (22.2)	48 (6.2)	
	Kidney problems	155 (18.2)	15 (18.5)	140 (18.2)	
	Eye problems	141 (16.6)	10 (12.3)	131 (17.0)	
	Do not know	335 (39.4)	25 (30.9)	310 (40.3)	
Diabetes mellitus has a genetic predisposition	Yes	371 (43.6)	39 (48.1)	332 (43.2)	0.004
	No	172 (20.3)	25 (30.9)	147 (19.1)	
	Don’t know	307 (36.1)	17 (21.0)	290 (37.7)	
There is a relationship between increased body weight and diabetes mellitus	Yes	549 (64.6)	53 (65.4)	496 (64.5)	<0.001
	No	129 (15.2)	28 (34.6)	101 (13.1)	
	Don’t know	172 (20.2)	0 (0.0)	172 (22.4)	
Smoking is a risk factor for diabetes mellitus	Yes	211 (24.8)	28 (34.6)	183 (23.8)	0.041
	No	214 (25.1)	13 (16.0)	201 (26.1)	
	Don’t know	425 (50.0)	40 (49.4)	385 (50.1)	
Diabetes is a controllable disease	Yes	708 (83.3)	55 (67.9)	653 (84.9)	<0.001
	No	142 (16.7)	26 (32.1)	116 (15.1)	
There is adequate awareness about diabetes in our society	Yes	535 (62.9)	46 (56.8)	489 (63.6)	0.228
	No	315 (37.1)	35 (43.2)	280 (36.4)	

**Figure 4:**
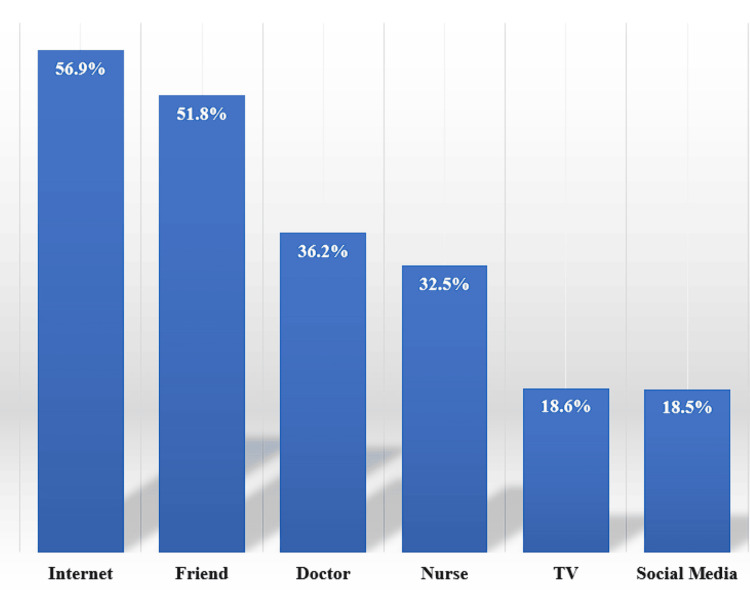
Source of information about diabetes mellitus among school students (n=850)

The findings demonstrated that, according to 56.9% of students, the internet served as the primary knowledge source for understanding DM, while friends came in second (51.8%). Only 36.2% and 32.5% of students acquired their information from doctors and nurses, respectively, while the least sources of knowledge were TV and social media, representing 18.6% and 18.5%, respectively (Figure 4).

## Discussion

The present study aimed to assess knowledge and attitude levels about DM among school students in the west of Saudi Arabia. Although Saudi Arabia is one of the Middle Eastern nations with the highest incidences of DM, there is a lack of knowledge about DM among Saudi school students [17,19]. The sample we looked at represents students at different educational levels before entering a university. This is a crucial time frame in which we can still affect the student's knowledge. However, after they proceed into higher education, they will rely on themselves to learn about diabetes and other health-related issues through the media, diabetic acquaintances, or family members.

The study's findings revealed that the majority of respondents (82.1%) were females, while only 17.9% were males, with the bulk of them aged 16-18. In contrast to this study, another survey conducted in Mecca found that male students (61.67%) outnumbered female students (38.33%). However, the later study found that the vast majority of participants (58.33%) were beyond the age of 16, which is consistent with our findings [18]. Male and young-age participants dominated a Jordanian study [20]. Overall, this study found that participants had limited knowledge about DM, which is similar to the results of previous studies in Saudi Arabia, Oman, and China, which indicated that school students had a general lack of understanding regarding DM [17,21,22]. This finding should be considered by policymakers and local authorities because, despite the development of information-dissemination channels such as the internet and social media, their levels of knowledge about common chronic diseases such as DM are still below expectations because there are not any school sessions or workshops on healthy eating and disease prevention.

According to numerous studies, up to 50% of T1DM risk factors are inherited [23], and first-degree relatives of those with T2DM are around three times more likely to contract the condition than people without a family history of the condition [24]. The current study results revealed that the percentage of self-reported DM among students was 9.5%, and the most prevalent type of diabetes was T1DM, which is similar to its prevalence among Saudi students in previous studies [25-27]. Among the diabetic students, we found that most of them (60.5%) had first-degree family members with diabetes. This was supported by another study from Oman, which stated that a family history of diabetes was reported by 62% of the students [21]. On the other hand, a study from Nigeria showed that only 11 out of 293 students had a family history of diabetes [28].

In this study, only half of the diabetic students followed their management plan, and 63% of them reported difficulties in their adherence to their antidiabetic medications. Studies that have been done in the past in Saudi Arabia support this [29-32]. The majority of a school-aged child's day is spent in school, which can provide substantial obstacles or serve as a great source of support for a child with diabetes. Therefore, school staff members should be better informed on DM and its management and provide aids for their students to improve their compliance with the DM management plan. About 75% and 66% of diabetic students reported that soft drinks and industrial juices increase their blood glucose level and make its control difficult, and this finding was proven before and mentioned in a meta-analysis run by Wang et al., who found that regular intake of sugary drinks increases the risk of T2DM and increases the body mass index [33].

About two-thirds of diabetic students suffer from psychological upset due to DM, and this was mentioned before in many studies that reported that DM patients are at significant risk for psychological, behavioral, and social issues because it is a chronic condition [34,35]. It is very important to care for the psychological health of such a group of vulnerable students through regular assessment and the provision of counseling sessions for them to be able to cope with such a chronic disease.

In contrast to a previous case-control study that demonstrated that both diabetic and non-diabetic adolescent groups had low rates of good eating habits, this study discovered that diabetic students followed a healthy diet at considerably higher rates than non-diabetic students [36]. This finding should be taken into consideration because a nutritious diet is vital for all pupils, not just those who are sick.

The highest proportion of students (38.4%) performed physical exercise such as walking or any other sport once or twice per week; 18.9% performed this exercise three to four days per week; and 12.8% of them performed it five days or more per week. Another study from China showed better results: 40% of the population claimed that they exercised more than three days per week [22]. These findings indicated that more clarification is required on the function of physical activity as a diabetes prevention strategy among school students. Adopting a healthy lifestyle, which includes a nutritious diet and regular exercise, is essential for the prevention and management of T2DM and has been linked to a lower chance of developing the disease [37]. Physical activity and exercise facilitate the uptake of glucose into body cells, improving insulin action and promoting glucose metabolism. Exercise can indirectly reduce the risk of DM by lowering body weight and decreasing insulin-resistant fat cells.

Regarding the knowledge of school students about DM, most of them (68.2%) knew that DM is a disease caused by a decreased insulin hormone secretion or a defect in its normal action that leads to increased plasma glucose levels. This result is consistent with the Omani study's findings, which showed that 52% of students properly defined DM [21]. However, their knowledge about its symptoms and complications was suboptimal, and only diabetic students could enumerate them. Therefore, it is very important to ask teachers to inform their students about this important issue. School health is a specialty of family medicine, and its role is to implement primary prevention to halt the uprising of diseases in children. Therefore, family physicians should provide frequent health education lectures for teachers, students, and parents to elevate their awareness levels about this important disease.

Obesity (64.4%), genetic predisposition (43.6%), and smoking (24.8%) were listed as risk factors for DM. The diabetic group of students showed significantly higher knowledge of these risk factors, with P values of 0.001, 0.004, and 0.041, respectively. Two studies that found that age (more than 40 years), overweight or obesity, high blood pressure, and smoking were all significant risk factors for DM provide support for this conclusion [21,38].

Our findings revealed that the internet was the primary source of information about DM. Another study from Oman verified this [21]. According to another survey, doctors (37%) and educators (33%) were the most prevalent sources of students' diabetes knowledge [28]. The disparities in rates could be related to variances in population race, age, cultural background, and availability of internet access, which has become a vital means of communication in Saudi Arabia within the last few years.

Limitations

Despite the fact that this study was done on a large sample that represents many geographic areas in the Saudi western region, there are a few restrictions that would need to be taken into consideration. First, this study is a self-reported one; therefore, we cannot ensure the actual prevalence of DM among this group of school students. Second, the non-probability approach to the selection of the students carries the threat of selection bias. Third, the sample's unequal gender stratification of students was one of the limitations of this study, as most research respondents were female students. This pitfall could be avoided by including it as an inclusion criterion for sample selection, allocating both genders proportionally based on the total number of students from both genders since we already reached more than double the calculated sample size.

## Conclusions

The students who contributed to this study had suboptimal levels of knowledge and attitude about DM. Moreover, the prevalence of DM was found to be high. Given the high prevalence of DM in western parts of Saudi Arabia, it is strongly advised to begin educating children about DM at a young age. Encourage students to participate in diabetes-related activities as well. School curricula should emphasize adopting healthy lifestyles among all school students with the availability of educational materials about healthy nutrition. As a result, it is highly suggested to perform screening for DM among children at high risk, specifically after we noticed the increasing prevalence of T2DM among young children. A number of studies should be carried out among school students and their parents or caregivers on a larger scale, and the information gathered should be used to create health programs aimed at educating children and their caregivers about healthy lifestyles, particularly proper nutrition for children of all ages and those in need, such as those with chronic illnesses such as DM. Focus groups with diabetic patients are used to conduct research on their psychological well-being in relation to chronic illnesses and to fully grasp the reasons why anti-diabetic medicine is not used as prescribed.
